# Hybrid Fabrication of Cold Metal Transfer Additive Manufacturing and Laser Metal Deposition for Ti6Al4V: The Microstructure and Dynamic/Static Mechanical Properties

**DOI:** 10.3390/ma17081862

**Published:** 2024-04-18

**Authors:** Zhenwen Chen, Yanning Liang, Cong Li, Xiaoyong Zhang, Jian Kong, Jikang Fan, Kehong Wang, Yong Peng

**Affiliations:** 1 School of Materials Science and Engineering, Nanjing University of Science and Technology, Nanjing 210094, China; chenzhenwen2012@163.com (Z.C.); lc1600@njust.edu.cn (C.L.); xiaoyong.zhang@njust.edu.cn (X.Z.); kongjian68@126.com (J.K.); fanjk@njust.edu.cn (J.F.); ypeng@njust.edu.cn (Y.P.); 2 Key Laboratory of Controlled Arc Intelligent Additive Manufacturing Technology, Nanjing University of Science and Technology, Nanjing 210094, China

**Keywords:** Ti-6Al-4V, CMT-LMD hybrid additive manufacturing, interface, tensile properties, dynamic compressive properties

## Abstract

The titanium alloy components utilized in the aviation field are typically large in size and possess complex structures. By utilizing multiple additive manufacturing processes, the precision and efficiency requirements of production can be met. We investigated the hybrid additive manufacturing of Ti-6Al-4V using a combination of cold metal transfer additive manufacturing (CMTAM) and laser metal deposition (LMD), as well as the feasibility of using the CMT-LMD hybrid additive manufacturing process for fabricating Ti-6Al-4V components. Microstructural examinations, tensile testing coupled with digital image correlation and dynamic compressive experiments (by the split Hopkinson pressure bar (SHPB) system) were employed to assess the parts. The results indicate that the interface of the LMD and CMTAM zone formed a compact metallurgical bonding. In the CMTAM and LMD zone, the prior-β grains exhibit epitaxial growth, forming columnar prior-β grains. Due to laser remelting, the CMT-LMD hybrid additive zone experiences grain refinement, resulting in equiaxed prior-β grains at the interface with an average grain size smaller than that of the CMTAM and LMD regions. The microstructures reveal significant differences in grain orientation and morphology among the zones, with distinct textures forming in each zone. In the CMT-LMD hybrid zone, due to interfacial strengthening, strain concentration occurs in the arc additive zone during tensile testing, leading to fracture on the CMTAM zone. Under high-strain-rate dynamic impact conditions, the LMD region exhibits ductile fracture, while the CMTAM zone demonstrates brittle fracture. The hybrid zone combines ductile and brittle fracture modes, and the CMT-LMD hybrid material exhibits superior dynamic impact performance compared to the single deposition zone.

## 1. Introduction

Ti-6Al-4V titanium alloy is commonly used as an engineering material due to its excellent mechanical properties, corrosion resistance, low density and biocompatibility. Therefore, it has been widely used in aerospace, aviation, marine engineering, biomedical and other fields [1,2,3,4]. Conventional manufacturing technologies present a lower material utilization and a higher manufacturing cost in forming highly complex geometric components. As a near-net shaping technology, additive manufacturing (AM) technology is better suited for designing and fabricating titanium alloy components [5,6,7]. Wire arc additive manufacturing (WAAM) and laser metal deposition (LMD) are two typical additive manufacturing (AM) technologies suitable for manufacturing large and complex components [8,9]. WAAM is a directed energy deposition (DED) technology utilizing the arc energy as the heat source, with advantages such as high deposition rates, high material utilization efficiency, low cost, and easy equipment operation, which is favorable for manufacturing large and complex titanium alloy structural parts in the aerospace and energy sectors [10,11,12].

CMT is a modified gas metal arc welding (GMAW) process that takes advantage of the digital control mode containing short arc and wire feeding monitoring commutation to realize a remarkable heat input reduction and better forming quality [13,14,15]. During the CMTAM process, it is capable of achieving less spatter, better arc stability and higher forming efficiency [16,17,18]. Lee used the He shielding gas to improve the Ti deposition quality by increasing the wettability through the effective arc energy in the CMT-GMA (cold metal transfer–gas metal arc) process of Ti-6Al-4V alloy deposition; the method improved the bead quality and increased the wire-feed deposition efficiency [19]. Lv improved the tensile property of cold metal transfer additive manufactured Ti-6Al-4V alloys via ultrasonic impact treatment; the strength and ductility were synchronously enhanced by double-sided UIT [20]. The disadvantage of the CMTAM process is that its forming precision is relatively lower than that of LMD, and the precision requirements for complex structural components are sometimes difficult to meet. LMD is a process that utilizes a laser source to melt metal powders delivered through a coaxial nozzle with carrier gas onto a metal substrate [21], and it has extensive advantages, including high formability rate when constructing complex features, small heat-affected zone, low deformation, high forming accuracy, strong adaptability to multi-material gradient formation and complex structures [22,23,24,25]. Extensive research on the microstructure and mechanical properties of LMD titanium alloys has been conducted. Choi applied LMD as a repair method for Ti-6Al-4V aerospace components and investigated the effect of two different deposition strategies and the resulting mechanical and fatigue properties for laser-deposited Ti-6Al-4V powder onto Ti-6Al-4V substrate [26]. Ma fabricated high-mass-proportion TiCp/Ti-6Al-4V composites with full density prepared by directed energy deposition, which significantly improved the hardness and wear resistance of titanium matrix composites [27].

The components used in the aviation field are often large in size and have complex structures. By using multiple additive manufacturing processes, the optimal processing technology can be selected according to the shape, size and requirements of the parts, thus improving the production flexibility in the manufacturing process [28,29,30]. Using multiple processes to manufacture parts can combine the advantages of each process and mitigate their shortcomings. This systematic approach helps address various challenges encountered in the manufacturing process, ultimately enhancing product quality [31,32,33,34]. In this study, CMTAM and LMD are combined and used for hybrid additive manufacturing of large and complex aerospace titanium alloy components. This approach not only fully leverages the advantages of CMTAM and LMD, but also meets the structural and performance requirements of new aerospace titanium alloy components. Due to the harsh operating environments and potential impact during flight, aerospace structural components have extremely strict performance requirements during their service life. Safety and stability are major issues that must be addressed. They must possess high enough strength and impact resistance to avoid dynamic damage failure under impacting loads [35,36,37]. When utilizing the hybrid additive manufacturing process of CMTAM and LMD to fabricate complex titanium alloy structural components, differences in microstructural organization and mechanical properties are observed due to the distinct heat source characteristics of each additive process. Currently, there is a dearth of meticulous investigation regarding the microstructure evolution and mechanical properties of multi-process hybrid additive titanium alloy components. The objective of this study is to employ the CMT-LMD hybrid process for Ti-6Al-4V component additive manufacturing and thoroughly analyze and investigate the microstructure as well as the static and dynamic mechanical properties across various sections of the CMT-LMD hybrid additive manufacturing Ti-6Al-4V titanium alloy components.

## 2. Experimental Details

### 2.1. Materials and Equipment

**Figure 1:**
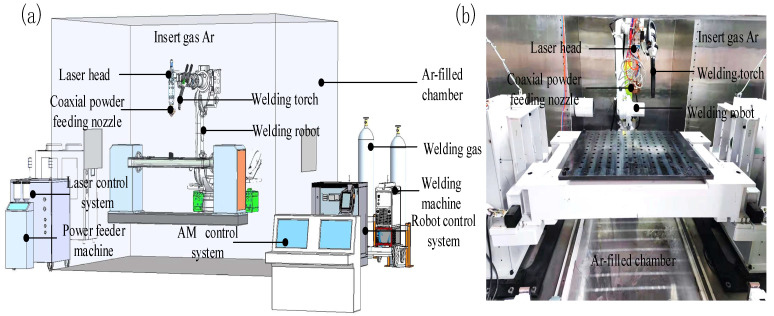
(**a**) Schematic of the CMTAM and LMD hybrid system and (**b**) overview of the CMTAM and LMD hybrid system.

The LMD parts were fabricated using an IPG YLS-6000 system (Marlborough, MA, USA), which consists of a 6 KW semiconductor laser, a pneumatic feeder, a four-coaxial-powder feeding head and a cooling-water machine. The CMT deposition system (Pettenbach, Tirol, Austria) consisted of a power source (CMT TPS5000), wire-feeding unit (VR 1500) and robotic torch system (Robata Twin Compact). The laser head and CMT welding torch were attached to an ABB Type IRB4600 Robot (Zürich, Switzerland) to provide accurate locations and movements. To prevent oxidation of the part during the manufacturing process, the entire deposition procedure was conducted in a 99.99% pure argon atmosphere, ensuring that the oxygen content remained below 50 ppm. The schematic and overview of CMT and LMD hybrid additive manufacturing equipment is shown in Figure 1a,b.

The deposition material chosen for the wire was Ti-6Al-4V (1.2 mm diameter). The Ti-6Al-4V powders used in the LMD process were produced by gas-atomized powder with a spherical shape, which were spherical particles with an average size about 53–150 μm. The chemical compositions of the wire and powder used in this study are presented in Table 1. The production of Ti-6Al-4V powders for use in the LMD process involves a plasma atomization process.

### 2.2. Manufacturing Process

**Figure 2:**
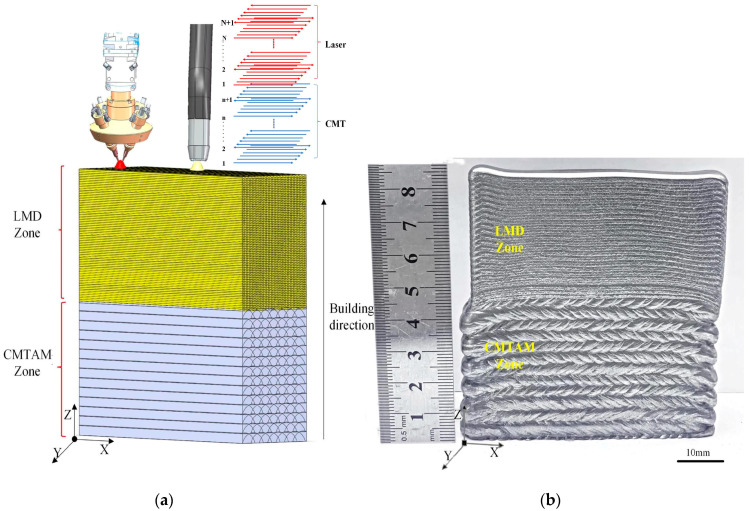
(**a**) Schematic drawing of the CMTAM and LMD hybrid process, schematic illustration of deposited sample geometry and deposition strategy and (**b**) photographs of the CMTAM and LMD hybrid sample.

As shown in Figure 2a, the component was fabricated by CMTAM and LMD, with a size of 80 × 85 × 30 mm, as shown in Figure 2b. CMT was adopted to finish manufacturing the bottom area of the sample; the main parameters are shown in Table 2. After the CMTAM part was finished, the robot switched to the LMD mode and continued to fabricate the upper area. The main experimental parameters of the laser melting deposition process are shown in Table 3. Each layer was deposited when the temperature of the previous layer was below 150 °C, which was measured by the infrared thermometer installed on the robot. As schematically illustrated, the deposition strategy for components fabricated by CMT and LMD methods both adopt the reciprocating alternating pattern.

### 2.3. Microstructure Characterization

**Figure 3:**
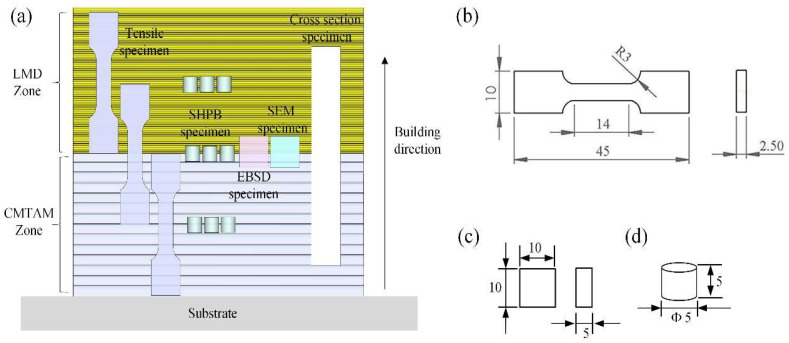
(**a**) Position of hybrid manufacturing microstructure characterization specimen and tensile and dynamic compressive sample, (**b**) size of tensile sample, (**c**) size of SEM and EBSD sample and (**d**) size of dynamic compressive sample.

As we can see from Figure 3, wire-cut electrical discharge machining (WEDM) was employed to obtain both metallographic specimens and EBSD samples from the connection area in the middle of the component. The microstructure was examined using optical microscopy (OM, Zeiss Smartzoom5) and scanning electron microscopy (SEM, FEI Quant 250FEG). Electron backscattered diffraction (EBSD) analysis was performed using an FEI Quattro ESEM operated at an acceleration voltage of 20 KV with a step size of 0.2 μm and a working distance of 18.5 mm. Prior to EBSD analysis, the EBSD sample underwent polishing to 4000 meshes using silicon carbide paper, followed by electrolytic polishing.

### 2.4. Mechanical Analysis

**Figure 4:**
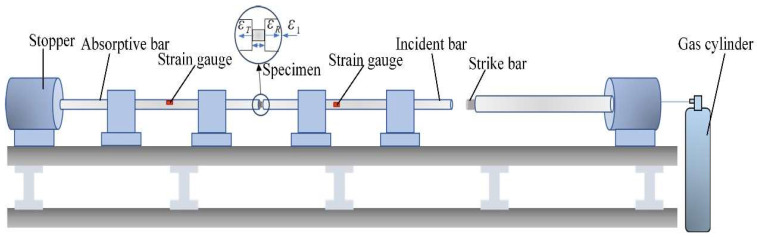
Schematic of the SHPB system.

In order to determine the tensile and dynamic properties of different zones, the CMTAM zone sample (C), the bonding zone sample (C + L) and the LMD zone sample (L) for uniaxial tensile testing and dynamic compression tests were taken as shown in Figure 3a. For each zone, three dog-bone-shaped specimens with gauge dimensions of 45 × 15 × 2.5 mm (Figure 3b) were tested for tensile strength at room temperature. The testing was conducted using an Instron-4403 testing machine at a constant displacement rate of 0.6 mm/min. The digital image correlation system (DIC) optical technique was used for the testing of different zones of CMT-LMD hybrid-processed samples to acquire a full strain data distribution with higher accuracy. Cylindrical samples (Φ5 × 5 mm) (Figure 3d) were obtained from different zones using wire electrical discharge machining. The axial direction of the samples was parallel to the deposition direction, as depicted in Figure 3a. The dynamic compressive experiments were carried out by the split Hopkinson pressure bar (SHPB) system (as seen in Figure 4). The dynamic compression tests were conducted at a temperature of 293 K with elevated strain rates ranging from 1900/s to 3200/s. To confirm the experimental data’s repeatability, at least three tests were carried out at each strain rate. SEM was utilized to examine the post-test specimens and gain insight into the failure mechanisms.

## 3. Results and Discussion

### 3.1. Initial Microstructure of the As-Built Sample

**Figure 5:**
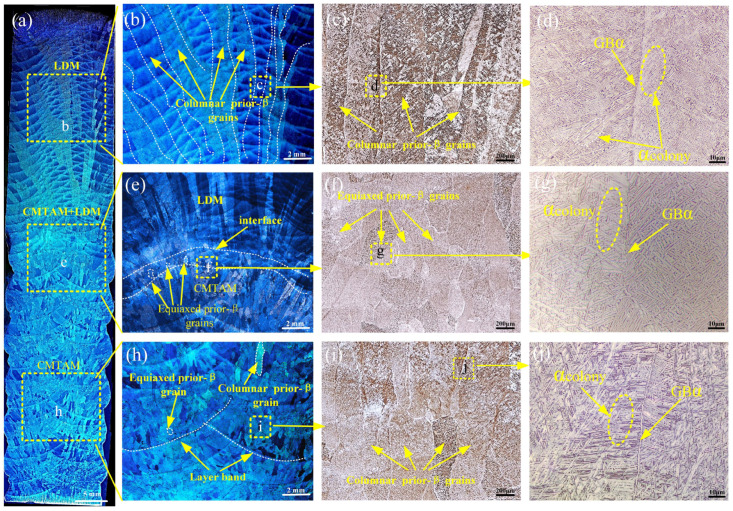
Macro morphology and microstructure of CMTAM and LMD hybrid-manufactured Ti-6Al-4V alloy: (**a**) CMTAM and LMD hybrid-manufactured Ti-6Al-4V alloy macrostructure, (**b**–**d**) LMD zone, (**e**–**g**) CMT-LMD hybrid zone and (**h**–**j**) CMTAM zone.

The macro cross-sectional views of the CMT-LMD hybrid additive sample in Figure 5a–j show that the interface of the LMD and CMTAM zone formed a compact metallurgical bonding. Powder LMD has higher precision and lower surface roughness compared with CMTAM. The optical micrographs of the LMD and CMTAM samples show a completely different microstructure, as shown in Figure 5. Based on different microstructural characteristics, the sample could be divided into three parts: LMD zone, CMT-LMD hybrid zone, and CMTAM zone. Significant variations in the morphology and size of the prior-β grains can be observed among these zones.

In the LMD zone, the heat input leads to the growth of these grains into the first few millimeters. With increasing build height, the prior-β grains become coarse columnar as a result of solidification and grow in a direction opposite to the heat flux, i.e., parallel to the build, and are decorated with a grain boundary α phase with an average width of 500–1100 µm. In the CMTAM zone, the prior-β grains consist of columnar and equiaxed grains, as shown in Figure 5i. The equiaxed prior-β grains are located at the bottom of each layer band, while the columnar prior-β grains are situated in the upper part of the layer bands. The width of the columnar prior-β grains is approximately 300–500 μm, with a length ranging from 1000 to 2000 μm. On the other hand, the equiaxed prior-β grains have a size of approximately 100–200 μm. In the CMT-LMD hybrid interface zone, the prior-β grains are equiaxed with dimensions of approximately 200 µm in length and width, which are smaller than the columnar prior-β grains in the CMT and LMD additive zones.

**Figure 6:**
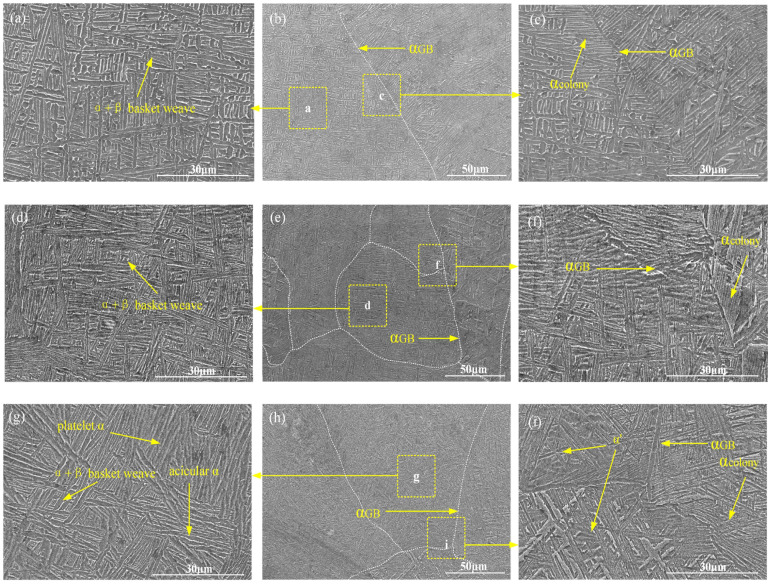
Microstructure of CMTAM and LMD hybrid manufacturing Ti-6Al-4V alloy: (**a**–**c**) LMD zone, (**d**–**f**) CMT-LMD hybrid zone and (**g**–**i**) CMTAM zone.

In Figure 5 and Figure 6, it can be observed that in the LMD zone, there is a continuous distribution of intergranular α phases along the prior-β grain boundaries. The width of GB-α (grain boundary α phase) is approximately 1 μm. The microstructure within the prior-β grains mainly consists of a basket-weave structure formed by partially aligned α plates and α colony distributed along the GB-α. The average length of the α phases is about 8 μm, with an average width of approximately 1.6 μm, and the aspect ratio is approximately 5, the sizes of the α phase and, grain boundary α phase in CMTAM and LMD hybrid- manufactured Ti-6Al-4V alloy are shown in Table 4.

In the CMTAM zone, there are mainly α martensite, basket-weave α + β, α colonies, and grain boundary α. Among them, the width of the GB-α phase is about 2 μm, and the average width of the α phase inside the prior-β grains is approximately 1.3 μm, with a length of about 22 μm. Based on this calculation, the aspect ratio is approximately 17.

In the CMT-LMD hybrid interface zone, the sizes of the α and β phases are relatively smaller compared to the CMTAM zone. The α/α’ phase further refines, and the needle-like α/α’ in this region is significantly shortened, forming a layered α structure and partial basket-weave α + β. The width of the GB-α is approximately 1 μm. The average width of the α phase inside the prior-β grains is about 0.7 μm, with a length of approximately 13 μm, resulting in an aspect ratio of 18.

Typically, the epitaxial growth of the prior-β grains in additive manufacturing titanium alloys is related to variations in thermal gradients and solidification conditions during the process. The thermal gradient, nucleation, and grain growth rates of the β grains are significantly influenced by the thermal input of the additive manufacturing process. The deposition of Ti-6Al-4V alloy occurs through solidification of a relatively “cooler” melt pool, which limits the applied thermal gradient and restricts the columnar growth of the prior-β grains. However, it can be argued that the higher thermal capacity and lower thermal conductivity of titanium alloys result in significant heat accumulation during the continuous layer deposition process. This leads to prolonged exposure of previously deposited layers to the temperature range that stabilizes the β phase, promoting epitaxial growth. Therefore, in the additive manufacturing process, the prior-β grains exhibit epitaxial growth, forming coarse prior-β grains.

As we can see from the bottom section of the CMTAM zone, the prior-β grains still exhibit epitaxial growth. The short-circuit transition in the CMTAM process effectively stirs the melt pool and partially restricts the epitaxial growth of the β phase, promoting grain refinement [38,39,40,41]. This is primarily attributed to the continuous oscillation of the wire feeder, which contributes to the stirring of the melt pool and causes the fragmentation of dendrites and grains from the mushy zone and partially melted region [42]. These fragmented particles are then carried into the melt pool, leading to the formation of more heterogeneous nucleation sites. Moreover, during the early stages of CMT deposition, the higher thermal gradients result in faster cooling rates, leading to a shorter duration of melt pool stirring and favoring the growth of dominant columnar prior-β grains. However, as the deposition height increases and heat accumulation gradually reduces the cooling rate, the stirring forces on the melt pool persist for longer periods. As a result, the early columnar β grains break, and nucleation occurs before the solidification front, resulting in the formation of more equiaxed β grains with increasing build height [43,44]. Therefore, at the top of the layer, the columnar prior-β grains are shorter, while at the bottom of the layer, some grains show equiaxed morphology. The aspect ratio of the α phase in the CMTAM process is greater than that in the LMD process.

When LMD is continued on the upper surface of the CMTAM zone, the top surface has already cooled to room temperature. At the top of the CMTAM zone, larger columnar grain boundaries of the prior-β are formed. When the laser melts the top CMTAM zone, the melt pool formed by the laser passing through the CMTAM zone remelts and solidifies, disrupting the previously formed coarse columnar grain boundaries of the CMTAM layer. At the same time, the surface of the arc layer affected by the laser has a higher cooling rate and temperature gradient, causing the growth direction of β grains to tend towards random growth, weakening the epitaxial growth effect. During the cooling process after the laser beam leaves, some equiaxed grains form at the end of the laser beam. Additionally, some grains continue to grow along the direction of the underlying coarse columnar crystals due to the influence of coarse columnar crystals in the lower layer [45]. In the interface region, the size of the prior-β grains and α phase are smaller compared to the CMTAM zone, mainly due to the high cooling rate and rapid solidification during the laser remelting of the CMT layer. When the laser beam left remelting of the CMT layer occurs, which increases the subcooling and leads to an increase in nucleation drive and nucleation rate, promoting grain refinement [46,47,48], and when the cooling temperature of the remelting region is below the β→α phase transformation temperature, a solid-phase transformation occurs, resulting in a finer prior-β structure compared to the CMTAM region. The size of the α phase is also related to the cooling rate: the size of the α phase, as well as the size of the α colony, decreases with increasing cooling rate.

### 3.2. Texture Evolution

**Figure 7:**
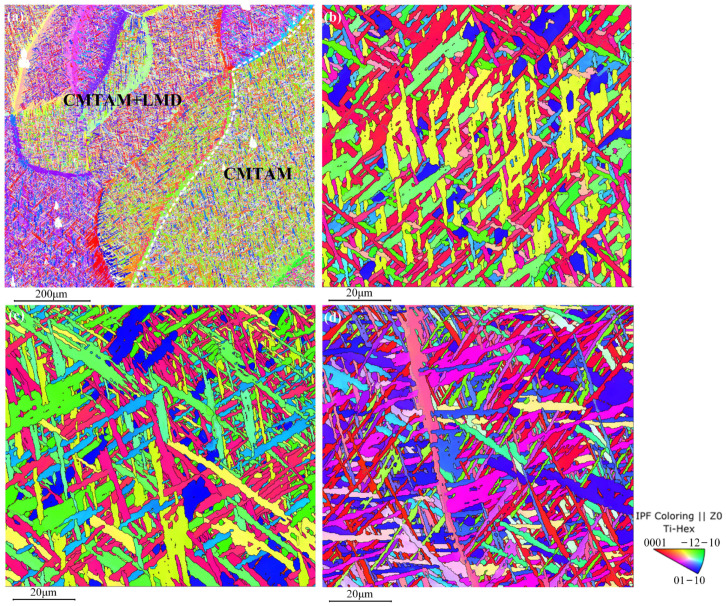
EBSD IPF image of CMTAM and LMD hybrid-manufactured Ti-6Al-4V alloy: (**a**) distribution of the β crystals, (**b**) LMD, (**c**) CMT-LMD and (**d**) CMTAM.

Figure 7a shows the hybrid zone of CMT-LMD, indicating a significant refinement and equiaxed morphology of the prior-β grains in the mixed region. The average size of the prior-β grains is 178 μm. Figure 7b–d represent the LMD zone, CMT-LMD hybrid zone, and CMTAM zone, respectively, as shown in the IPF (inverse pole figure) images. Similar to the microstructures depicted in Figure 6, the IPF images reveal significant differences in grain orientation and morphology among the various zones. A strong texture is mainly formed on {0001} during LMD sequential printing, which is consistent with the direction of the maximum cooling rate, as shown Figure 7b. A strong texture is mainly formed on {−12–10} in the CMT-LMD zone. A strong texture is mainly formed on {01–10} in the CMTAM zone.

**Figure 8:**
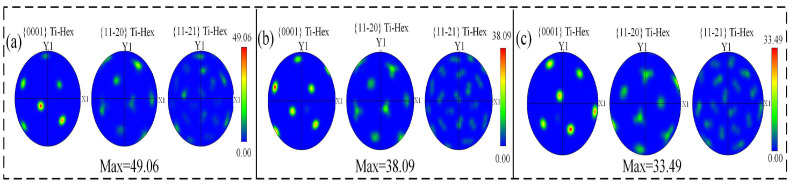
EBSD pole figure of specimens: (**a**) LMD, (**b**) CMT-LMD and (**c**) CMTAM.

A significant discrepancy in the texture between the struts fabricated by the two different approaches is concluded from the corresponding pole figures in Figure 8a–c. The maximum polar density of the LMD zone is 49.06, of the CMT-LMD zone is 36.45 and of the CMTAM zone is 33.49. The maximum polar density of LMD zone is significantly higher than those of the CMT-LMD zone and CMTAM zone. The presence of a high polar density in the metal material texture can lead to anisotropy in the mechanical properties. Laser remelting and recrystallization will affect the structure of titanium alloy and texture strength.

**Figure 9:**
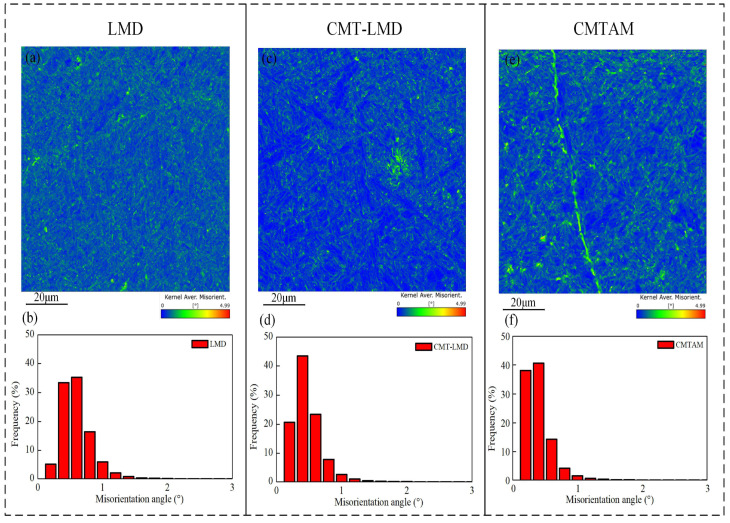
EBSD KAM map and misorientation angle distribution map of different zones (**a**,**b**) LMD, (**c**,**d**) CMT-LMD and (**e**,**f**) CMTAM.

Figure 9 shows the KAM for different zones. KAM is typically calculated quantitatively to determine the geometric dislocation density and reflect the level of plastic deformation uniformity. Higher numerical values in the map indicate regions with more significant plastic deformation or higher defect density. KAM values are visually depicted using color bands, wherein the color blue represents the minimum value and red represents the maximum value. The range of KAM values is within the 0° to 5° range. Upon observing the KAM diagram for the CMTAM zone, it becomes evident that the proportion of green color is relatively prominent, indicating higher KAM values. In turn, this indicates a lower dislocation density within the zone.

### 3.3. Uniaxial Tensile Behavior

**Figure 10:**
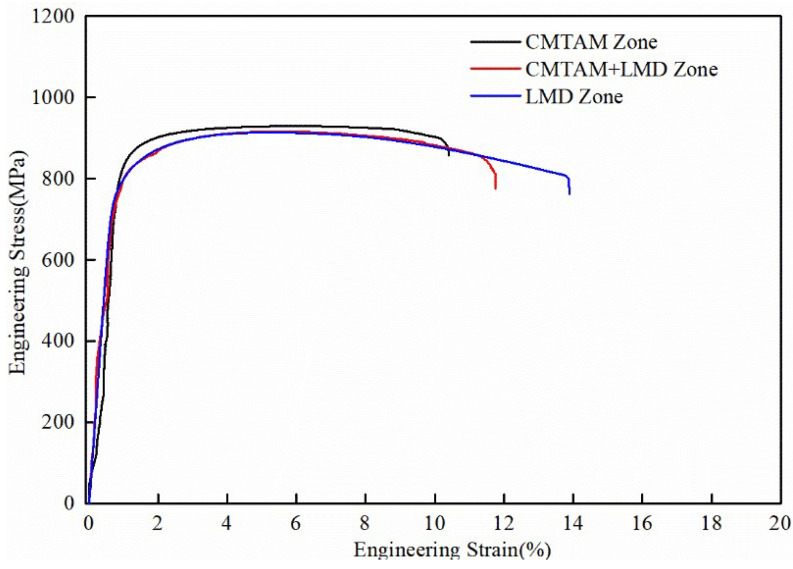
Tensile stress–strain curves of CMT-LMD hybrid-processed samples.

Through tensile testing of specimens from different zones, as shown in Figure 10’s stress–strain curve and Table 5, it can be observed that the ultimate tensile strength (UTS) of the CMTAM zone is 929 MPa, with a yield strength (YS) of 828 Mpa and a ductility level of 10.4%. The CMT-LMD hybrid zone exhibits an ultimate tensile strength of (UTS) 915 Mpa, a yield strength (YS) of 803 Mpa and a ductility level of 11.7%. The LMD zone shows an ultimate tensile strength (UTS) of 913 Mpa, a yield strength (YS) of 801 Mpa and a ductility level of 13.9%. The CMT-LMD hybrid zone displays comparable tensile strength to the LMD zone, while the CMTAM zone exhibits slightly higher strength than both the LMD zone and the CMT-LMD zone. Additionally, the ductility level of the CMT-LMD region falls between those of the CMTAM and LMD zones.

**Figure 11:**
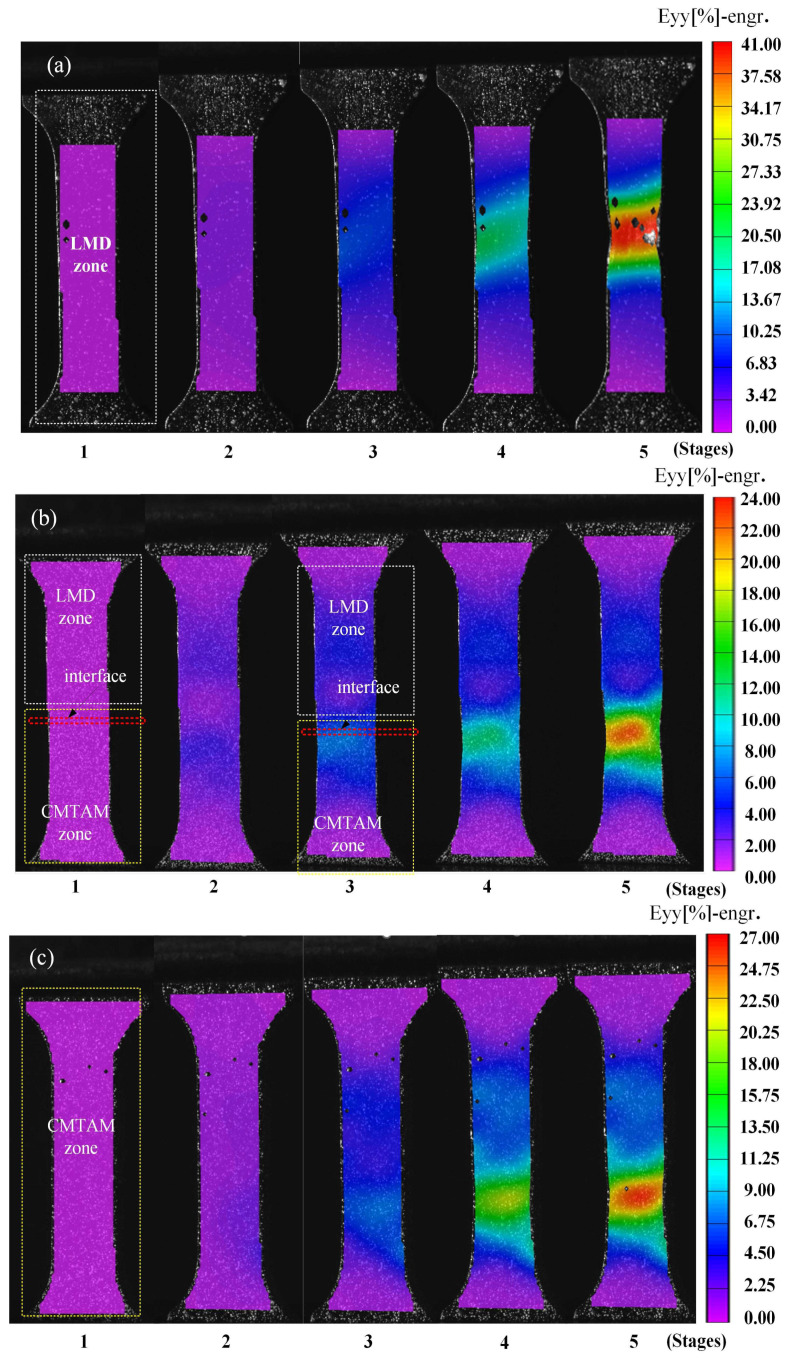
Contour maps of DIC in different zones of CMT-LMD hybrid-processed samples during tensile testing: (**a**) LMD, (**b**) CMT-LMD and (**c**) CMTAM. 1, 2, 3, 4, 5 correspond to loading times of 0, 1.5, 3, 4.5 mins and the imminent fracture under the deformation rate of 0.6 mm/min, respectively.

Figure 11 shows the axial strain distributions visualized by DIC of different zones of the CMT-LMD hybrid-processed samples during tensile testing. With an increase in the tensile load, non-uniform deformation and strain concentration were observed in all three groups of specimens. Additionally, necking occurred in the high-strain region. The sample of the LMD zone exhibited a greater concentration of strain, while the sample of the CMTAM zone demonstrated a more uniform distribution of strain. In the sample of the CMT-LMD hybrid zone, during the initial loading phase, strain concentration occurred in both the laser additive area and the arc additive area, while no strain concentration was observed at the interface between the two processes. However, as stress increased, strain concentrated in the CMTAM region and eventually led to fracture in the CMTAM region.

**Figure 12:**
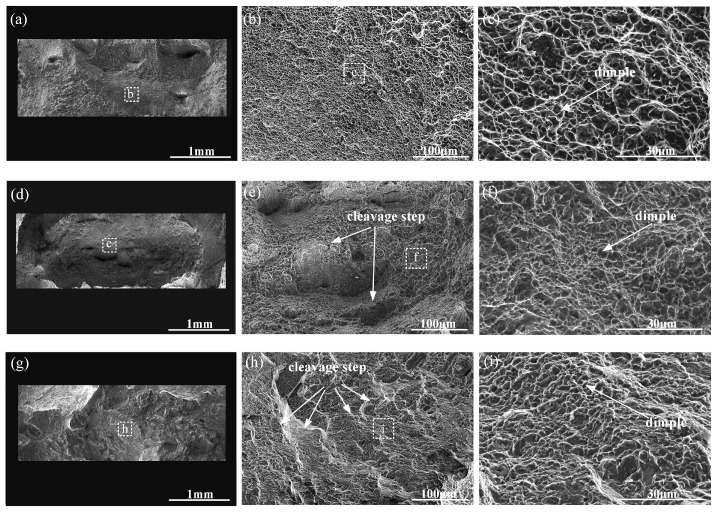
SEM images of the fracture surface of tensile samples in different zone: (**a**–**c**) LMD, (**d**–**f**) CMT-LMD and (**g**–**i**) CMTAM.

Figure 12 shows the SEM images of the fracture surface of tensile samples in different zones. It can be seen from Figure 12a–c that the LMD zone exhibits a significant number of large and deep dimples after the fracture of the tensile specimens, indicating that it is capable of undergoing substantial deformation under load and possesses good ductility and energy absorption capacity. The LMD zone is primarily composed of a basket-weave α + β structure. Previous studies have found that the fine laths of the LMD zone with different orientations have short slip distances and confine the space between gliding dislocations, and the probability of dislocation annihilation is reduced, thereby enhancing both the stored dislocation density and the capacity for strain hardening. Moreover, the presence of fine laths in the LMD microstructure effectively inhibits stress concentration and strain localization, thereby delaying the initiation of micro-cracks and micro-voids. During non-uniform deformation, the fine microstructure is also beneficial to reducing the impingement of slip bands on grain boundaries and phase boundaries, which will increase the resistance to the initiation and propagation of cracks and lead to a ductile fracture mechanism, Therefore, the LMD zone exhibits high plasticity in terms of tensile mechanical properties.

The fracture surface of the CMT tensile specimen exhibits cleavage steps. During the propagation of cleavage cracks, these steps merge with each other, forming river-like patterns, as shown in Figure 12g–i. The presence of brittle and ductile regions can result in a ductile–brittle mixed fracture behavior. In the columnar primary β grains of the of CMTAM zone, the main constituents are α’ martensite and lamellar α phase. α’ martensite, as a phase transformation product, is typically formed in titanium alloys through rapid cooling or under stress. It exhibits high hardness and brittleness and can significantly enhance the strength of titanium alloys. However, it simultaneously reduces their ductility. When stress is applied to the titanium alloy, martensitic crystals are prone to dislocation slip and crack propagation, resulting in limited plastic deformation and low ductility. The sample exhibits relatively high strength but limited ductility. Therefore, it has the highest strength and lowest ductility in tensile testing.

**Figure 13:**
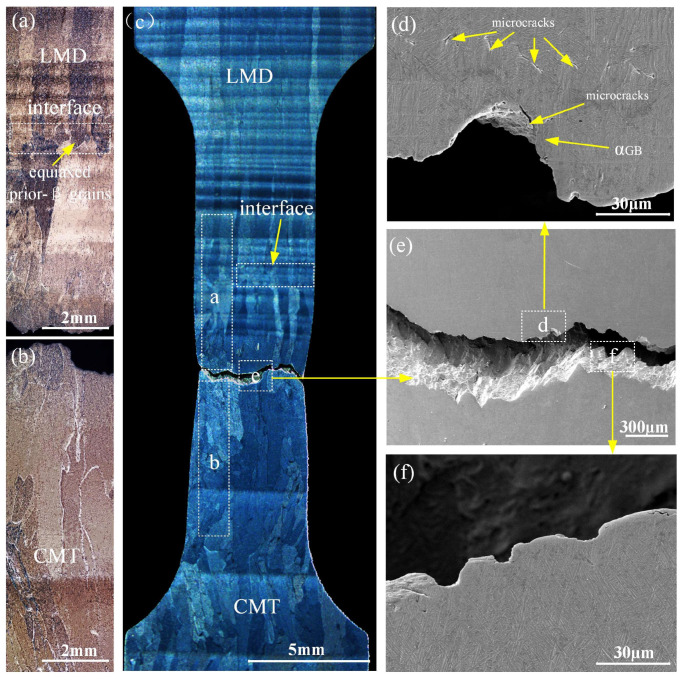
Images of fracture cross-sections of the tensile tested specimens: (**a**,**b**) OM image of fracture cross-sections, (**c**) fracture microstructure of hybrid-processed samples and (**d**–**f**) SEM images of fracture cross-sections of the tensile tested specimen.

From Figure 13a–c, it can be observed that the fracture of the CMT-LMD hybrid zone tensile specimen occurs in the CMTAM region. As shown in Figure 12d–f, the fracture morphology of the CMT-LMD hybrid region is similar to that of the CMTAM region, showing cleavage steps and shallow, small dimples, indicating that the CMT-LMD region also belongs to a mixed fracture mode. Furthermore, from Figure 13c, it can be seen that there is significant necking during the tensile process in the CMT-LMD zone, and the specimen fractures in the CMTAM zone. Therefore, the elongation of the hybrid-formed sample mainly reflects the performance of the CMTAM zone, which exhibits relatively low ductility. As shown in Figure 13d–f, the main crack propagates along the coarse prior-β grain boundaries and α colony, and short micro-cracks can be observed along or through the α lamellae in the microstructure, with a length of approximately 22 ± 3 μm. The laser remelting of the CMTAM zone not only disrupts the formation of coarse columnar grain boundaries and refines the prior-β grains but also results in the decomposition of α martensite into α + β in the interface region.

According to the Hall–Petch relationship [49,50], the grain refinement at the interface leads to an increase in interfacial strength. Consequently, the performance of the laser-arc interface region is higher than that of the CMTAM region, which explains why no fracture occurred in the interface region.

During the tensile test, the non-uniformity of the prior-β grain structure results in strain concentrating on weak areas of the sample. Due to the presence of grain boundaries, dislocations generated during the tensile process accumulate at the grain boundaries, leading to crack initiation in the internal weak regions. In this study, the yield strength and elongation of the Ti-6Al-4V hybrid-manufactured sample mainly depend on the mechanical properties of the CMTAM. The prior microstructural feature of the CMTAM sample is the growth of columnar grains along the deposition direction, and its mechanical tensile performance is significantly affected by the angle between the tensile load direction and the direction of columnar grain growth [45]. When the tensile load is perpendicular to the growth direction of the prior columnar grains, the presence of irregular dislocations promotes the formation of micro-cracks along the grain boundaries, leading to relatively poor tensile performance.

### 3.4. Dynamic Mechanical Properties

#### 3.4.1. Dynamic Stress–Strain Response

**Figure 14:**
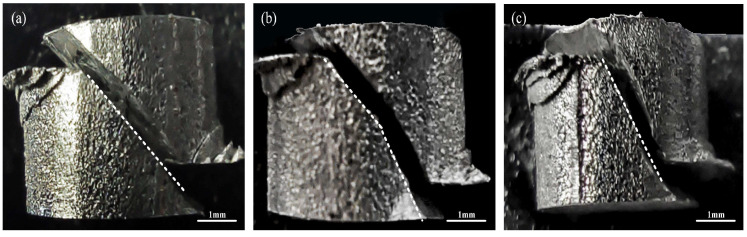
Macroscopic samples after dynamic impacts in different zones at a strain rate of 3200/s: (**a**) LMD zone, (**b**) CMT-LMD zone and (**c**) CMTAM zone.

In this study, the dynamic properties of different zones (LMD, CMT-LMD, CMTAM) were tested by a split Hopkinson pressure bar (SHPB). The strain rates were 1900/s, 2600/s, and 3200/s, respectively. When the loading strain rates were 1900/s and 2600/s, no visible cracks were observed on the surface of the cylindrical specimens, and no failure occurred. The specimens underwent plastic deformation under uniaxial compressive stress, with an increasing degree of reduction in height as the loading strain rate increased. However, when the loading strain rate was increased to 3200/s, we can see in Figure 14 that macroscopic cracks appeared on the cylindrical specimens, resulting in obvious failure. The macroscopic morphology of the specimen after deformation and cracking at 3200/s showed significantly different paths of crack propagation in different regions.

**Figure 15:**
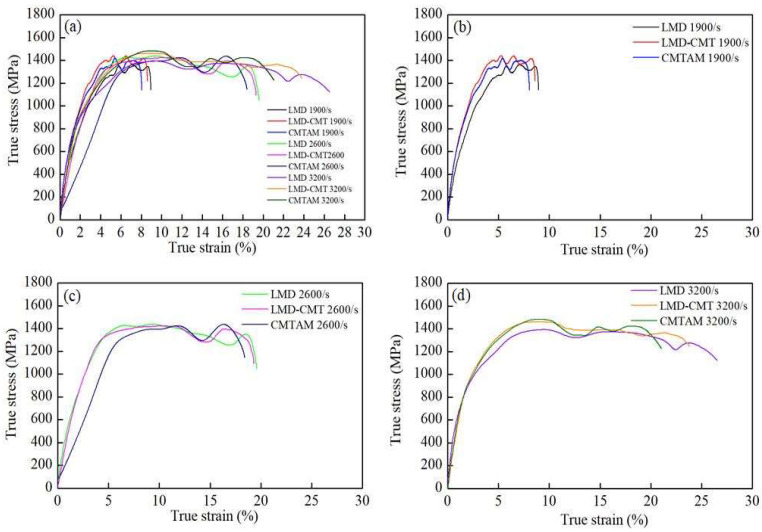
Dynamic stress–strain curves from SHPB tests at strain rates ranging from 1900 to 3200/s for different zones: (**a**) 1900 to 3200/s for LMD, CMT-LMD, CMTAM; (**b**) 1900/s; (**c**) 2600/s; and (**d**) 3200/s.

From Figure 15 and Table 6, it can be observed that the true strain gradually increases with the increase in strain rate for all zones of the specimens. Under non-destructive loading conditions (1900/s and 2600/s), the dynamic plastic strain in the three regions increases with increasing strain rate. Under destructive loading conditions (3200/s), the strength shows an increase, exhibiting a certain strain hardening effect. The ultimate strength and yield strength of the LMD zone specimens are slightly lower than those of the CMT-LMD hybrid zone and CMTAM zone within the entire range of strain rates, while the true strain is higher than those of the above two zones. The CMTAM zone exhibits the highest ultimate strength and the least plastic deformation. Under strain rates of 1900/s and 2600/s, the CMT-LMD hybrid zone shows comparable ultimate strength to CMTAM and comparable plastic deformation to the LMD zone. At a strain rate of 3200/s, its ultimate strength is slightly lower than that of the CMTAM zone, and its plastic deformation is slightly lower than that of the LMD zone, demonstrating good dynamic mechanical performance overall.

#### 3.4.2. Fracture Morphology

**Figure 16:**
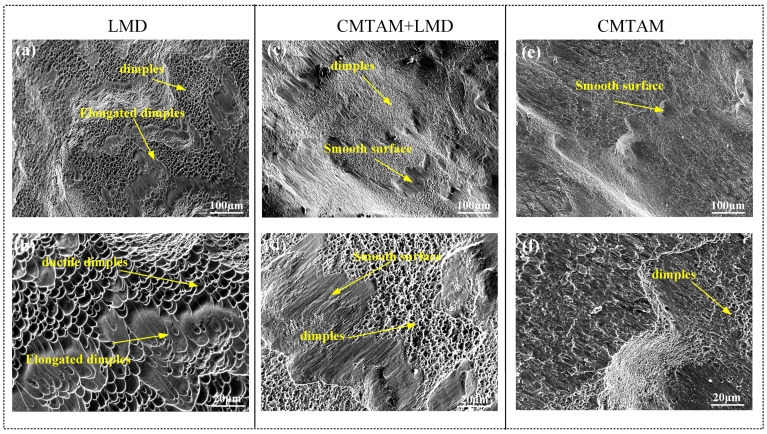
Fracture morphologies of different zones: (**a**,**b**) LMD zone, (**c**,**d**) CMT-LMD zone and (**e**,**f**) CMTAM zone.

Figure 16 shows the dynamic compression fracture surface morphology, where the central region of the fracture was selected for SEM observation and analysis. In the LMD samples, numerous pits and a few cleavage fractures are observed (Figure 16a,b). The sample of the CMT-LMD zone displays a substantial number of pits with a few cleavage steps (Figure 16c,d). Additionally, the CMTAM zone exhibits more cleavage fractures and some dimples. At high magnification, the LMD samples exhibit deep and large pits and elongated dimples due to shear forces, while the dimples in the CMTAM samples are shallow and small (Figure 16e,f). The CMT-LMD surface exhibits a mixed morphology of now-faded and smooth regions, with densely distributed dimples of certain depth.

During the dynamic impact process, a significant amount of work is required to induce plastic deformation in titanium alloys due to their high strength. In this process, the majority of the work is transformed into heat. However, due to the poor thermal conductivity of titanium alloys, a large amount of heat cannot dissipate quickly, resulting in a significant increase in temperature close to or reaching its melting point. When the deformation process is disturbed by minor perturbations, the combined effects of the aforementioned factors lead to a rapid amplification of the disturbances, ultimately forming an adiabatic shear band and causing material failure [51,52].

When the high temperature within the shear band softens the material, the relative motion and friction between the two fracture surfaces of the specimen cause severe deformation or even disappearance of some dimples in the direction of shear stress, forming a smooth region. The dimples generated along the shearing direction exhibit significant orientation, indicating intense plastic deformation. Dimple regions are formed by the nucleation, growth and ultimate connection of micropores, exhibiting characteristics of ductile fracture [53,54,55]. Therefore, based on the dimples on the fracture surface, it can be observed that the LMD and CMT-LMD samples exhibit a ductile fracture mode, while the CMTAM sample shows a brittle fracture mode, consistent with the true stress–strain curve shown in Figure 15.

Furthermore, according to Table 5, it can be observed that the LMD and CMTAM samples have different plasticity and strength. The dynamic compression specimens used in this study form bi-layered composite structures with distinct microstructure and properties. Ti-based layered structures have better ballistic performance than homogeneous ones [56,57]. A high-performance metallic alloy with a good combination of strength and ductility is achieved through cold metal transfer and laser hybrid additive manufacturing. During the high-strain dynamic impact process, the CMT-LMD hybrid zone exhibits uneven plastic deformation. The CMTAM zone, which has poor ductility, serves as a weaker link, while the LMD zone, which has better plasticity, serves as a support layer. It absorbs a significant portion of the impact energy and resists crack propagation. Therefore, it exhibits good dynamic mechanical properties during high-strain dynamic impact.

## 4. Conclusions

This study investigated the tensile and dynamic impact behavior of Ti-6Al-4V alloy specimens fabricated by cold metal transfer and laser hybrid additive manufacturing. The microstructure, tensile properties, dynamic compression performance and deformation and fracture mechanisms were comprehensively studied. The following conclusions can be drawn:

(1) The interface between the LMD and CMTAM zones showed significant grain refinement due to laser remelting, consisting mainly of fine equiaxed prior-β grains, with a microstructure of lamellar α and basketweave α + β inside the grains.
(2) The LMD zone exhibited higher texture intensity compared to the CMTAM and the CMT-LMD hybrid zone, indicating significant anisotropy. The CMTAM zone and the CMT-LMD hybrid zone had higher dislocation densities than the LMD zone.
(3) The interface between the hybrid additive manufacturing specimens exhibited good bonding and tensile mechanical properties. During the tensile testing process, fracture occurs in the CMTAM zone due to interface strengthening caused by remelting at the interface between CMTAM and LMD.
(4) With the increase in the strain rate, the dynamic compressive strength and total strain increased for all specimens of the CMTAM, LMD and CMT-LMD hybrid zones. High-performance structures with a good combination of strength and ductility were achieved through cold metal transfer and laser hybrid additive manufacturing, and the CMT-LMD hybrid additive manufacturing zone displayed a higher energy absorption capacity during high-strain-rate dynamic impact.

## Figures and Tables

**Table 1:** Element composition of Ti-6Al-4V wire and powder.

Element	Al (wt%)	V (wt%)	Fe (wt%)	C (wt%)	N (wt%)	O (wt%)	Ti (wt%)
Ti-6Al-4V Wire	5.96	4.07	1.5	0.0082	0.0068	0.085	Bal.
Ti-6Al-4V Powder	6.06	4.04	0.03	0.009	0.009	0.057	Bal.

**Table 2:** Main experimental parameters of CMT gas metal arc deposition process.

Wire Feeding Rate v2/(m·min^−1^)	Mean Current U/V	Mean Current I/A	Gas Flow Rate Q/(L·min^−1^)	The Interpass Temperature/°C
4.8	19	150	20	≤150 °C

**Table 3:** Main experimental parameters of laser melting deposition process.

Laser Power P/KW	Overlap Ratio%	Scanning Speed v1/(mm·s^−1^)	Powder Feeding Rate v2/(g·min^−1^)	Spot Diameter mm	The Interpass Temperature/°C
1.8	50	10	7.5	2.7	≤150 °C

**Table 4:** The sizes of the α phase and grain boundary α phase in CMTAM and LMD hybrid-manufactured Ti-6Al-4V alloy.

Part	α phase			GBα
	Width/μm	Length/μm	Aspect Ratio	Width
LMD	1.6	8	5	1
CMTAM-LMD	0.7	13	17	1
CMTAM	1.3	22	18	2

**Table 5:** Mechanical properties obtained from stress–strain curves. (Yield strength (YS), ultimate tensile strength (UTS), elongation (EL)).

Sample	YS (Mpa)	UTS (Mpa)	EL (%)
LMD	801 ± 0.7	913 ± 3.1	13.9
CMT-LMD	803 ± 2.1	915 ± 2.6	11.7
CMT	828 ± 3.1	929 ± 3.2	10.4

**Table 6:** Dynamic mechanical properties obtained from dynamic stress–strain curves.

Sample	True Stress/MPa	True Strain/%	Strain Rate (/s)
LMD1	1364	8.94	1877
LMD2	1389	19.6	2618
LMD3	1396	26.6	3263
CMT-LMD1	1426	8.61	1863
CMT-LMD2	1441	19.3	2639
CMT-LMD3	1465	23.9	3217
CMTAM1	1421	8.1	1917
CMTAM2	1438	18.4	2626
CMTAM3	1481	21	3228

## Data Availability

Data is unavailable due to privacy or ethical restrictions.
